# Students’ Willingness to Intervene in Bullying: Direct and Indirect Associations with Classroom Cohesion and Self-Efficacy

**DOI:** 10.3390/ijerph15112577

**Published:** 2018-11-17

**Authors:** Sebastian Wachs, Ludwig Bilz, Saskia M. Fischer, Wilfried Schubarth, Michelle F. Wright

**Affiliations:** 1 Department of Educational Studies, University of Potsdam, 14476 Potsdam, Germany; wilschub@uni-potsdam.de; 2 Brandenburg University of Technology Cottbus-Senftenberg, Institute of Health Sciences, 01968 Senftenberg, Germany; ludwig.bilz@b-tu.de (L.B.); saskia.fischer@b-tu.de (S.M.F.); 3 Department of Psychology, Pennsylvania State University, PA 16802, USA; mfw5215@psu.edu; 4 Faculty of Social Studies, Masaryk University, 60200 Brno, Czech Republic

**Keywords:** bullying, intervention, willingness to intervene, verbal bullying, relational bullying, aggression, school, classroom climate, classroom cohesion, self-efficacy

## Abstract

Although school climate and self-efficacy have received some attention in the literature, as correlates of students’ willingness to intervene in bullying, to date, very little is known about the potential mediating role of self-efficacy in the relationship between classroom climate and students’ willingness to intervene in bullying. To this end, the present study analyzes whether the relationship between classroom cohesion (as one facet of classroom climate) and students’ willingness to intervene in bullying situations is mediated by self-efficacy in social conflicts. This study is based on a representative stratified random sample of two thousand and seventy-one students (51.3% male), between the ages of twelve and seventeen, from twenty-four schools in Germany. Results showed that between 43% and 48% of students reported that they would not intervene in bullying. A mediation test using the structural equation modeling framework revealed that classroom cohesion and self-efficacy in social conflicts were directly associated with students’ willingness to intervene in bullying situations. Furthermore, classroom cohesion was indirectly associated with higher levels of students’ willingness to intervene in bullying situations, due to self-efficacy in social conflicts. We thus conclude that: (1) It is crucial to increase students’ willingness to intervene in bullying; (2) efforts to increase students’ willingness to intervene in bullying should promote students’ confidence in dealing with social conflicts and interpersonal relationships; and (3) self-efficacy plays an important role in understanding the relationship between classroom cohesion and students’ willingness to intervene in bullying. Recommendations are provided to help increase adolescents’ willingness to intervene in bullying and for future research.

## 1. Introduction

Bullying in schools is a prevalent problem and can have serious physical, mental, social, and behavioral short- and long-term consequences for those involved [1,2,3]. Bullying is defined as any repeatedly aggressive behavior against persons or groups that cannot readily defend themselves [4]. Bullying may involve physical assault, such as hitting or kicking, verbal harassment, such as hurtful name-calling, verbal threats, and verbal abuse, as well as selectively undermining social relationships through manipulation or exclusion from social groups [5]. There are a range of different roles that adolescents can take in bullying situations, that differ from the commonly described roles of bully, victim, and bully-victim. “Assistants”, for instance, join the bullies, “reinforcers” provide positive feedback to bullies (e.g., cheering), but do not actively assist the bullies, “outsiders” withdraw from bullying situations, “defenders” side with the victims and support them, and “bystanders” see the bullying occur but do not choose a side, thereby, passively enabling bullying to continue [6]. The most common way that people experience bullying is observing it as a bystander [7]. The tendency for bystanders to remain passive might be explained by, for instance, their lack of a sense of responsibility for helping the victim, fearing being judged unfavorably by peers for helping the victim, not realizing that the situation is perceived as uncomfortable, being afraid that they could also be bullied if they get involved, and lacking the skills to intervene in bullying behaviors [8]. Bystanders have received a lot of attention by scholars, because research has shown that bullying prevention and intervention programs that emphasize and encourage bystanders to defend victims result in a decrease in bullying overall and an increase the success of bullying interventions [9,10]. In addition, some research suggests that if bystanders become actively involved in bullying, on the side of the victim, then they are often successful in ending the bullying in that particular situation and they can also have positive impact on the victims’ adjustment and social status [11,12]. There are additional reasons to empower bystanders to give up their passive role in bullying. There is some evidence, for instance, that witnessing bullying can affect the bystanders’ mental health, school satisfaction, and increase their levels of stress and negative feelings (e.g., anxiety) [13,14,15]. Possible explanations for these findings might include, the cognitive dissonance they experience when faced with the discrepancy between their lack of action and their desire to intervene; a sense co-victimization; and feelings of powerlessness [13]. As there is some evidence that other students seldom intervene to defend the victims of bullying [16,17,18], it is important to understand which factors increase students’ willingness to intervene. Such findings might help to deepen our knowledge about successful bullying prevention and intervention in schools and help us protect bystanders from experiencing negative psychological and school-related outcomes, when faced with bullying.

According to Bandura [19], self-efficacy is defined as one’s “beliefs in one’s capabilities to organize and execute the courses of action required to produce given attainments.” (p. 3). The conviction that it is possible to cope through one’s action with what is required of one socially, even under difficult conditions, is an essential motivational foundation in individuals and an important predictor of socially-competent behavior in childhood and adolescence [20]. In addition, students with high self-efficacy are more likely to challenge themselves with difficult tasks and recover faster from setbacks [19]. Students with low self-efficacy, on the other hand, often shy away from challenging tasks, because they view these tasks as threats rather than challenges that they can overcome [19]. It is possible that a domain-specific form of self-efficacy might be important in students’ willingness to stand up to bullying, because if they lack confidence in their capacity to successfully intervene, they would be less likely to act [21]. The positive association between students’ perceived self-efficacy, in terms of actually or potentially intervening in bullying, has been highlighted in a number of studies, using qualitative and quantitative methods, self-reports and peer nominations, and cross-sectional and longitudinal study designs [21,22,23,24,25,26,27].

There are a number of factors, along with self-efficacy, that play a crucial role in whether students do decide to defend bullying victims or not. From a socio-ecological perspective [28], the microsystem, which consists of structures or locations where individuals have direct interactions (i.e., family, peer group, school, and school class), has immediate influence on the active or passive behavior of bystanders [17,29,30]. In Germany, students in the fifth and sixth grade usually stay in the same classroom with the same students, during the school day. Therefore, it seems reasonable to consider the subjective perceived peer relationship, within a class, as a possible correlate of students’ willingness to intervene in bullying. Indeed, previous research showed that a positive school/classroom climate which also includes aspects, such as classroom cohesion, is positively correlated with students’ readiness to intervene in bullying [31,32,33].

Classroom cohesion understood as one facet of classroom climate, however, might not only be associated with students’ willingness to intervene in bullying but also with students’ self-efficacy toward intervening in bullying. In Bandura’s view [19], self-efficacy affects one’s behaviors and the social environments with which people are interacting, and is influenced by one’s actions and significant others, such as parents, siblings, teachers, and peers. Particularly, during adolescence, peer acceptance and relationships are important to the adolescents’ development of self-efficacy because peers contribute considerably to adolescents’ socialization and views of themselves [34,35]. Peers provide adolescents with possibilities to learn from each other and function as role models. Thus, it can be assumed that in classrooms that are characterized by strong cohesion and mutual sympathy among classmates, students meet the right conditions to develop self-efficacy. Indeed, initial research found a positive link between the classroom student–student relationship quality and students’ self-efficacy towards bullying [33].

To summarize, previous research attempted to elucidate the personal and contextual factors that explain students’ willingness to intervene in bullying situations. These studies have found that the defender’s self-efficacy [21,22,23,24,25,26,27] and school climate [31,32,33] might play a crucial role. There is also some evidence to suggest that school climate (i.e., cohesion) is related to students’ defender self-efficacy beliefs [33]. Therefore, the present study aims to investigate whether the relationship between school climate and willingness to intervene in bullying might be mediated by self-efficacy. We expect that school climate will increase self-efficacy, which in turn increases students’ readiness to intervene in bullying. More specifically, we hypothesized:

**Hypothesis 1** **(H1).** Classroom cohesion will be directly associated with greater self-efficacy, and students’ willingness to intervene in bullying situations.

**Hypothesis 2** **(H2).** Classroom cohesion will be indirectly associated with students’ willingness to intervene in bullying situations via greater self-efficacy.

## 2. Materials and Methods

### 2.1. Participants and Sampling Procedure

This study uses data from a stratified random sample (strata: three types of school) in the Eastern German federal state of Saxony. A randomized probability-proportional-to-size sampling scheme was used, where the size is equivalent to the number of students per school. There were forty-one schools initially contacted for inclusion in this study, but twenty-four agreed to participate (roughly a 60% response rate at school level). A maximum of six classes per school were asked to participate in the study, resulting in three classes in the sixth grade and three classes in the eighth grade. Only those students within these classes were invited to participate in the study.

The final sample included 2071 participants (51.2% males; *n* = 1060), in the sixth grade (*n* = 1080) and eighth grade (*n* = 991), from 114 classes across 24 schools. Of the schools, seven were secondary grammar schools, thirteen were non-academic-track secondary schools, and four schools were for children with special needs (students with emotional and behavioral disorders, and students with learning disabilities). Overall, 581 students were not included in the study because they did not have written parental consent (*n* = 419), had a doctor’s note excuse on the days of data collection (*n* = 109), had an unexcused absence from school (*n* = 2), were absent because of school projects (*n* = 26), were attending internship (*n* = 4), refused to participate (*n* = 8), were on vacation (*n* = 4), and were new to the class and uninformed about the study (*n* = 2).

Age of participants ranged from 12 to 17 years (*M* = 13.63, *SD* = 1.17). The age breakdown of participants is as follows: 20.6% (*n* = 427) were 12 years old, 27.1% (*n* = 562) were 13 years old, 23.8% (*n* = 493) were 14 years old, 24.6% (*n* = 509) were 15 years old, 3.3% (*n* = 69) were 16 years old, 0.3% (*n* = 6) were 17 years old, and 0.2% (*n* = 5) did not specify their age. Most of the students (50.4%; *n* = 1044) attended non-academic-track secondary schools, followed by 43.7% (*n* = 904) for secondary grammar schools, and 5.9% (*n* = 123) who attended schools for children with special educational needs. Most participants were born in Germany, with 1.9% (*n* = 40) who were not born in Germany. Demographic characteristics of participants, organized by grade, sex, and type of school are displayed in Table 1.

### 2.2. Measures

#### 2.2.1. Students’ Willingness to Intervene in Bullying Situations

The questionnaire started with a definition of traditional bullying that outlined the central characteristics of bullying (intention to hurt, imbalance of power, and repetition), as postulated by Olweus [4], in order to increase the validity of responses. The vignettes used to assess students’ willingness to intervene in bullying was adopted from Yoon and Kerber [36]. The first vignette assessed students’ willingness to intervene in verbal bullying situations:

“You hear a sixth-grade student calling out to another: “Nerd, bootlicker, asshole”. The student tries to ignore the remarks but is obviously sad. You have already observed the same thing a few days ago”.

The second vignette asked for students’ willingness to intervene in relational bullying situations:

“During the break, you hear a sixth-grade student say to another: “No, absolutely not. I’ve already told you that you can’t join us.” The student has no friends in class and is alone throughout the break and looks sad. It’s not the first time that the student was excluded from playing”.

After each vignette, participants were asked to rate how much they agree with the following statement: “When I observe something like that, I interfere and try to stop the behavior”. The participants could choose from five responses—(1) “completely disagree”, (2) “disagree”, (3) “neither agree nor disagree”, (4) “agree”, and (5) “completely agree”.

#### 2.2.2. Self-Efficacy in Social Conflicts

The scale is intended to capture self-efficacy expectations when dealing with social demands and conflicts. The scale consists of three items like, “I manage to cope well even with difficult classmates” [37]. A high value expresses the conviction of a person to act competently in conflictive situations. Participants could choose between four responses—(1) “completely disagree”, (2) “disagree”, (3) “agree”, and (4) “completely agree”. Cronbach’s alpha was 0.68.

#### 2.2.3. Classroom Cohesion

To assess the extent of cohesion and mutual sympathy among students in a class, as one facet of classroom climate, a scale consisting of three items was used, including items such as “When someone says something against our class, everyone sticks together” [38]. Participants could then choose between five responses—(1) “completely disagree”, (2) “disagree”, (3) “neither agree nor disagree”, (4) “agree” and, (5) “completely agree”. Cronbach’s alpha was 0.71.

#### 2.2.4. Socio-Demographic Variables

Socio-demographics were assessed by asking for information on students’ grade (6th or 8th), age, sex (1 = male or 2 = female), and type of school (non-academic-track secondary school (Oberschule), school for students with special needs (Förderschule), and grammar school (Gymnasium)). Previous research has shown significant differences regarding sex and age in students’ willingness to intervene in bullying, with girls compared with boys and younger students compared with older students more likely to defend [17,18,21,25,39]. Hence, sex, and age were included as control variables.

### 2.3. Procedures

The data protection officer and educational authority of the federal state of Saxony in Germany approved the study. After approval, email invitations were sent to the forty-one schools. Data were collected from June 2014 to October 2014, using paper-pencil questionnaires that were administered by trained research assistants. Signed written parental consent was obtained from children who were minors. During data collection, participants were informed that their participation was voluntary and anonymous, and that they could stop participating in the study at any time, without any penalty. The total time to administer the questionnaire was 30–45 min and it was administered during normal school hours.

### 2.4. Data Analyses

To begin with, the mean scores were computed by averaging items on the questionnaires. Then correlation analyses and descriptive statistics were used to investigate the main study variables. In a next step, mediation analysis was conducted in structural equation modeling framework with classroom cohesion, self-efficacy, and students’ willingness to intervene in bullying, as latent variables. The latent variable “willingness to intervene in bullying” was composed of the willingness to intervene in a verbal bullying incident and the willingness to intervene in a relational bullying incident. The factor loadings of these two observed variables were freely estimated, so the factor variance of the latent factor was fixed to one. Mediation analysis was used to test direct effects of classroom cohesion on self-efficacy, in social conflicts, and students’ willingness to intervene in bullying, as well as indirect effects of classroom cohesion on students’ willingness to intervene in bullying, via self-efficacy in social conflicts. The mediation analysis was completed in Mplus 8.0 software (Muthén & Muthén, Los Angeles, CA, USA) [40]. Since the dependent variables, willingness to intervene in verbal bullying, and willingness to intervene in relational bullying, departed from normality, in their distribution, maximum likelihood estimation with robust standard errors (MLR) was used for the analyses. The significance of indirect effects was assessed by using a bias-corrected bootstrapping procedure, with 5000 samples. Full information maximum likelihood (FIML) estimation was used to address missing data issues. To account for the multilevel structure of the data (i.e., students nested within school classes) standard errors were corrected by using the complex sampling option in Mplus (complex-option).

## 3. Results

### 3.1. Descriptive Statistics

Regarding students’ willingness to intervene in verbal bullying, 20.9% (*n* = 428) of adolescents answered that they ‘completely disagree’, 22.4% (*n* = 458) ‘disagree’, 34.9% (*n* = 716) ‘neither agree nor disagree’, 16.4% (*n* = 337) ‘agree’, and 5.4% (*n* = 110) ‘completely agree’ to intervene. Similarly for students’ willingness to intervene in relational bullying, 22.6% (*n* = 463) of adolescents answered that they ‘completely disagree’, 25.3% (*n* = 518) ‘disagree’, 33.4% (*n* = 684) ‘neither agree nor disagree’, 13.4% (*n* = 274) ‘agree’, and 5.2% (*n* = 107) ‘completely agree’ to intervene. Correlations between the study variables, means, and standard deviations are shown in Table 2. All correlations were in the expected direction. Higher levels of classroom cohesion were positively correlated with higher levels of self-efficacy in social conflicts, and willingness to intervene in bullying. Self-efficacy was positively correlated with willingness to intervene in bullying. There were also some significant correlations between the control and the main study variables. Girls, in comparison with boys, reported higher levels of willingness to intervene in bullying. With increasing grade, adolescents reported lower levels of willingness to intervene in verbal and relational bullying.

### 3.2. Direct and Indirect Associations between Classroom Cohesion, Self-Efficacy, and Students’ Willingness to Intervene in Bullying

**Figure 1:**
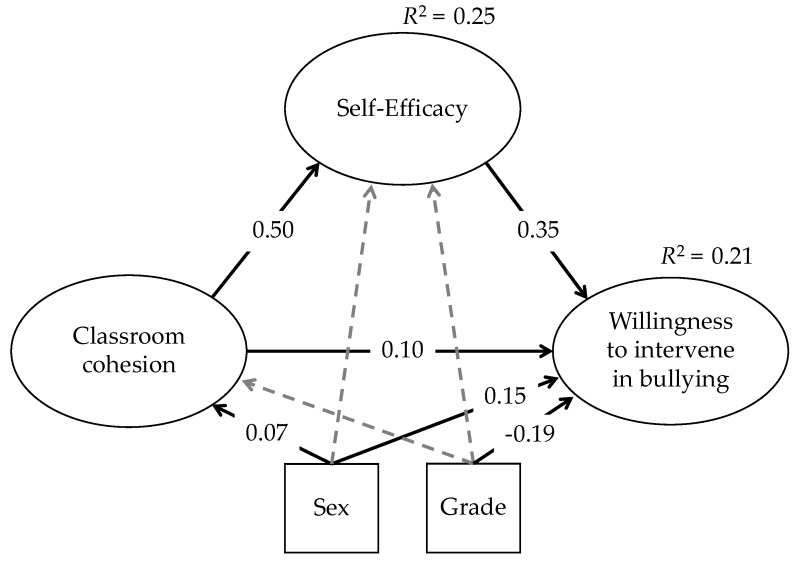
Direct and indirect effects of the latent variables classroom cohesion, self-efficacy, control variables, and willingness to intervene in bullying. Notes: Dash arrows—non-significant path coefficients.

We investigated the direct effects of classroom cohesion on self-efficacy in social conflicts and students’ willingness to intervene in bullying, as well as indirect effects of classroom cohesion on students’ willingness to intervene in bullying, via self-efficacy in social conflicts. The model had an excellent fit: χ^2^ = 56.58 *df* = 27 χ^2^/*df* = 2.09, *p* < 0.001, comparative fit index (CFI)= 0.99, Tucker–Lewis index (TLI) = 0.98, root mean square error of approximation (RMSEA) = 0.02 (90% CI = 0.01, 0.03), standardized root mean square residual (SRMR) = 0.02. As shown in Figure 1, classroom cohesion had a direct effect on self-efficacy in social conflicts (β = 0.50, *p* < 0.001), as well as on students’ willingness to intervene in bullying (β = 0.10, *p* < 0.001). There was also a direct effect of self-efficacy in social conflicts on students’ willingness to intervene in bullying (β = 0.35, *p* < 0.001). The indirect effect of classroom cohesion on students’ willingness to intervene in bullying, via self-efficacy in social conflicts, was also significant (β = 0.18, *p* < 0.001). The analyses also revealed significant effects of the control variables on classroom cohesion and students’ willingness to intervene in bullying. Girls scored significantly higher than boys on the measures of students’ willingness to intervene in bullying (β = 0.15, *p* < 0.001), as well as on classroom cohesion (β = 0.07, *p* < 0.001). Additionally, an increasing grade was negatively associated with students’ willingness to intervene in bullying (β = −0.19, *p* < 0.001). Other paths in the model did not reach statistical significance. The proposed model explained 21% of variance in students’ willingness to intervene in bullying (*R*^2^ = 0.21) and 25.5% of variance in self-efficacy in social conflicts (*R*^2^ = 0.25). Finally, we analyzed the mediation model, separately, by grade (6th vs. 8th) and sex (female vs. male), and did not find any relevant grade or sex differences in the direct and indirect effect sizes. These results can be requested from the first author.

## 4. Discussion

The present study aimed to investigate the direct and indirect associations between classroom cohesion, self-efficacy in social conflicts, and students’ willingness to intervene in bullying, among German students. Several interesting findings emerged. 

A first important finding of the present study was that personal and contextual factors need to be considered, in order to better understand students’ readiness to intervene in bullying situations. We found support for our prediction that self-efficacy in social conflicts and classroom cohesion would be directly associated with students’ willingness to intervene in bullying situations (H1). The analyses also showed that the effect of self-efficacy in social conflicts (β = 0.35) on students’ intervention willingness was nearly the same as the effect of classroom cohesion (total effect: direct effect, β = 0.10 + indirect effect, β = 0.18) on students’ intervention willingness. This finding further underlines that both predictors are nearly equally important to explain students’ willingness to intervene in bullying. Overall, our findings are consistent with previous research revealing the relationships between students’ willingness to intervene in bullying and self-efficacy [21,22,23,24,25,26,27], and school climate [31,32,33]. Self-efficacy regarding social conflicts might be vital for understanding students’ willingness to intervene in bullying. Support for this proposal is found in previous research suggesting that students with high self-efficacy in social conflicts are less inhibited when intervening in bullying, are more likely to tackle difficult tasks, and recuperate much quicker from setbacks [19,20,21]. Positive peer relationships might influence students’ readiness to intervene because classroom cohesion characterized by positiveness, mutual respect, and warmth, might be a solid fundament for positive peer group norms. In these classes, rejection and exclusion of single students might be less tolerated and respectful interaction promoted. Taken together, these findings suggest that efforts to address students’ willingness to intervene in bullying should involve measures to encourage interpersonal relationships and support within the school classes, and students’ efficacy to deal with social conflicts.

We add valuable knowledge to the literature on bystander behavior in bullying, regarding the indirect association between classroom cohesion and students’ willingness to intervene in bullying via self-efficacy, in social conflicts. Hence, we found support for our hypothesis that classroom cohesion would be indirectly associated with students’ willingness to intervene in bullying, via greater self-efficacy in social conflicts (H2). This finding was consistent for different age groups (6th vs. 8th graders) and for boys and girls, as shown by differential analyses of grade and sex. A possible explanation for the indirect effect might be that students learn from their peers how to intervene from each other and that some peers function as role models to students, within a class. Indeed, vicarious experience has been shown to be an important source of self-efficacy [19]. Thus, observing peers successfully intervening in bullying may also contribute to enhancing self-efficacy, in social conflicts such as bullying. Intervention and prevention programs to increase students’ intervention willingness should aim to foster positive relationships within the school class. This might positively influence students feeling to be efficacious towards social conflicts, which in turn increases the willingness for intervention in bullying. It is worth mentioning that the indirect effect of classroom cohesion on students willingness to intervene in bullying (β = 0.18) was larger than the direct effect of classroom cohesion on students willingness to intervene (β = 0.10). This means that a substantial amount of the effect between classroom cohesion and students’ willingness to intervene can be explained by self-efficacy. Our findings also highlight the important role of classroom cohesion for self-efficacy, in social conflicts, in intervening in bullying among adolescents, which has also been shown by previous research on the development of general self-efficacy [34,35] and initial research on students’ defending behavior [33].

Another noteworthy result was that nearly half of the students reported that they would not intervene in verbal (43%) and relational bullying (48%) situations. The finding that students often did not believe they would intervene, is in accordance with previous research from other countries [16,17,18]. Reasons to remain passive might be the absence of feeling responsible for helping the victim, fearing unfavorable judgment by peers when helping the victim, not realizing that the situation is perceived as uncomfortable, being afraid that they could also be bullied if they get involved, and the lack of skills to intervene in bullying behaviors [8]. Regarding sex and grade differences, we found that girls were more likely to intervene in bullying situations, when compared to boys, and with increasing grades, students were less likely to intervene in bullying situations. These differences are also similar to what has been reported by past research [17,18,21,25,39]. However, previous research found girls to have higher levels of moral sensitivity and lower moral disengagement than boys [21]. In addition, girls are more likely than boys to interpret bullying situations as emergencies and report bullying to an adult and ask for social support, which can increase their readiness to stand up for the victims [41,42]. On the one hand, the grade differences found are somewhat counterintuitive because with increasing age social and cognitive skills are further developed [23]. On the other hand, the results are in line with past research that showed that bullying increases among students between 11 to 13 years old [8]. Possible explanations for the grade differences might be that younger adolescents might underestimate the potentially negative social consequences of interventions, whereas older students are more reluctant as they believe that interventions could make them the next target and might seem like they are violating peer norms [43]. In addition, with increasing age, students believe that victims should be able to solve their problems by themselves and, therefore, they do not want to solidarize with the socially-stigmatized victims, often against a powerful, perceived bully, within the peer network [44]. Finally, the students in our sample were 12 to 15 years old. This might have limited the possibilities to draw any conclusion for age differences regarding intervention willingness.

Taken together, it appears to be important to raise awareness among students that they can be successful to end bullying and that their actions could have a positive impact on the victims’ adjustment. Particularly, boys and older students need to be addressed in intervention prevention measures to increase their willingness to intervene in bullying. However, the findings of the present study highlight that awareness is most likely not a sufficient condition. Students need also the right social environment, characterized by positive peer-peer relationships and the confidence that they are capable to deal with bullying, efficaciously.

## 5. Limitations and Outlook on Future Research

First, in the present study we measured behavioral intentions, rather than actual intervention behavior. This means, we assessed how students believe they would intervene in a given situation, but not necessarily how they have actually intervened. To overcome this shortcoming, future studies should measure actual intervention behavior by asking students to recall a specific bullying situation and how they behaved in that situation. Second, the present study relies exclusively on self-reports to assess bystander behavior. Therefore, social desirability might be a concern because students might tend to hide their true behavior about not helping the victim. Follow-up research should try to replicate the findings of the present study by using peer-nominations or a combination of peer- and self-reports. Third, because of the cross-sectional design of this study, it is not possible to determine the temporal ordering among classroom cohesion, self-efficacy, and students’ willingness to intervene in bullying. Follow-up studies should include a longitudinal study design with at least three measurement points to further substantiate the mediating relationships tested in the present study. Fourth, we assessed only one facet of classroom climate, namely cohesion, and we used a general measurement of self-efficacy in social conflicts and not a specific self-efficacy scale concerning bullying, both of which might have deflated the estimates. Future studies should try to replicate our findings by using a multifaceted-scale, for measuring classroom climate, and a domain-specific self-efficacy scale which measures students’ readiness to intervene in bullying. In addition, both scales showed relatively low reliabilities, as measured by Cronbach’s alpha. Finally, in addition to verbal and relational bullying scenarios, used in the present study, future research should include physical and cyber bullying and compare students’ potential or actual intervention behavior, among a wider range of student bullying behavior. Finally, future studies could also investigate whether self-efficacy and classroom cohesion play the same role in sexual, racial, or homophobic harassment situations.

## 6. Conclusions

To understand which factors increase students’ willingness to intervene in bullying might deepen current knowledge concerning the success of bullying prevention and intervention in schools, and protect bystanders from experiencing adverse health and school-related outcomes. The present study revealed that self-efficacy in social conflicts and classroom cohesion were directly associated with students’ willingness to intervene in bullying situations. Furthermore, this study was one of the first to examine the indirect associations among classroom cohesion, self-efficacy in social conflicts, and students’ willingness to intervene in bullying. Findings indicate that the more positive the students’ perceived peer-to-peer relationships within the classroom, the more efficacious students felt towards social conflicts, and the more likely they were to intervene in bullying situations. This finding was consistent for different age groups and girls and boys. Another finding was that a high percentage of students reported that they would never or rarely intervene in bullying, with boys and older students being less likely to intervene. Thus, we conclude that (1) it appears crucial to increase students’ motivation to intervene in bullying; (2) efforts to address students’ willingness to intervene in bullying should promote students’ confidence in dealing with social conflicts and interpersonal relationships within school classes; and (3) self-efficacy plays an important role in understanding the relationship between classroom cohesion and students’ willingness to intervene in bullying.

## Figures and Tables

**Table 1:** Frequencies of demographic variables by grade, sex, and type of school.

Grade	Sex	Type of School							
		Grammar Schools		Non-Academic-Track Secondary School		Schools for Children with Special Needs		Total	
		*n*	%	*n*	%	*n*	%	*n*	%
6th grade	Boys	230	25.4	312	30.1	33	26.8	575	27.8
	Girls	250	27.7	215	20.6	37	30.1	502	24.3
8th grade	Boys	194	21.5	258	24.8	33	26.8	485	23.5
	Girls	230	25.4	255	24.5	20	16.2	505	24.4
Total		904	43.7	1040	50.3	123	5.9	2067 *	100

Note. * Four participants did not specify their sex.

**Table 2:** Bivariate correlations between classroom cohesion, self-efficacy, intervention willingness, and control variables, means, and standard deviations.

Variables	1	2	3	4	5
1. Intervention willingness	–				
2. Self-efficacy in social conflicts	0.29 **	–			
3. Classroom cohesion	0.14 **	0.26 **	–		
4. Grade ^8th grade^	−0.10 **	0.04 *	0.01	–	
5. Sex ^female^	0.07 **	0.03	0.01	0.04 *	–
Mean	2.66	2.81	3.21	–	–
*SD*	0.96	0.57	0.88	–	–

* *p* < 0.05. ** *p* < 0.01.
